# Naturally-Occurring Invasive Urothelial Carcinoma in Dogs, a Unique Model to Drive Advances in Managing Muscle Invasive Bladder Cancer in Humans

**DOI:** 10.3389/fonc.2019.01493

**Published:** 2020-01-21

**Authors:** Deborah W. Knapp, Deepika Dhawan, José A. Ramos-Vara, Timothy L. Ratliff, Gregory M. Cresswell, Sagar Utturkar, Breann C. Sommer, Christopher M. Fulkerson, Noah M. Hahn

**Affiliations:** ^1^Department of Veterinary Clinical Sciences, Purdue University, West Lafayette, IN, United States; ^2^Purdue University Center for Cancer Research, Purdue University, West Lafayette, IN, United States; ^3^Department of Comparative Pathobiology, Purdue University, West Lafayette, IN, United States; ^4^Department of Oncology and Urology, Sidney Kimmel Comprehensive Cancer Center, Johns Hopkins University School of Medicine, Baltimore, MD, United States

**Keywords:** animal models, bladder cancer, cancer prevention, dog, immunotherapy, targeted therapy, transitional cell carcinoma, urothelial carcinoma

## Abstract

There is a great need to improve the outlook for people facing urinary bladder cancer, especially for patients with invasive urothelial carcinoma (InvUC) which is lethal in 50% of cases. Improved outcomes for patients with InvUC could come from advances on several fronts including emerging immunotherapies, targeted therapies, and new drug combinations; selection of patients most likely to respond to a given treatment based on molecular subtypes, immune signatures, and other characteristics; and prevention, early detection, and early intervention. Progress on all of these fronts will require clinically relevant animal models for translational research. The animal model(s) should possess key features that drive success or failure of cancer drugs in humans including tumor heterogeneity, genetic-epigenetic crosstalk, immune cell responsiveness, invasive and metastatic behavior, and molecular subtypes (e.g., luminal, basal). Experimental animal models, while essential in bladder cancer research, do not possess these collective features to accurately predict outcomes in humans. These key features, however, are present in naturally-occurring InvUC in pet dogs. Canine InvUC closely mimics muscle-invasive bladder cancer in humans in cellular and molecular features, molecular subtypes, immune response patterns, biological behavior (sites and frequency of metastasis), and response to therapy. Thus, dogs can offer a highly relevant animal model to complement other models in research for new therapies for bladder cancer. Clinical treatment trials in pet dogs with InvUC are considered a win-win-win scenario; the individual dog benefits from effective treatment, the results are expected to help other dogs, and the findings are expected to translate to better treatment outcomes in humans. In addition, the high breed-associated risk for InvUC in dogs (e.g., 20-fold increased risk in Scottish Terriers) offers an unparalleled opportunity to test new strategies in primary prevention, early detection, and early intervention. This review will provide an overview of canine InvUC, summarize the similarities (and differences) between canine and human InvUC, and provide evidence for the expanding value of this canine model in bladder cancer research.

## Introduction

Urinary bladder cancer (urothelial carcinoma, also referred to as transitional cell carcinoma) is a major human health issue worldwide with more than 400,000 new cases per year (1, 2). Broadly speaking, human bladder cancer can be divided into two general types. The more common form which comprises approximately two thirds of cases, consists of low-grade non-invasive cancer that can typically be managed with transurethral resection and intravesical therapy (3). Although this cancer is manageable, it can negatively impact the patients' quality of life, frequently recurs, and can progress to invasive cancer (3). The less common, but more serious form of bladder cancer consists of high-grade muscle-invasive urothelial carcinoma (InvUC). InvUC is associated with a 50% lethality rate, marked reduction in quality of life from the cancer and its treatment (cystectomy, radiation therapy, chemotherapy), and high medical costs ($150,000 to >$200,000 per patient) (4–10). In the last two to three decades, only modest improvement has occurred in the outcome of patients with InvUC. This review will focus on this challenging form of bladder cancer, InvUC.

Encouraging progress has recently been made in new therapies aimed at molecular, epigenetic, and immune targets in InvUC (10–12). The finding of differential treatment responses based on molecular InvUC subtypes (luminal, basal, etc.) along with combinations of these new drugs, is expected to lead to dramatic improvements in InvUC therapy (11–18). There are, however, insufficient numbers of patients to test even a fraction of the new drugs, especially when considering various possible drug combinations, in order to optimize therapy. The numbers of patients with metastases who could still be eligible for trials after failing standard of care therapies are especially limited. This puts higher demands on pre-clinical animal studies to identify the most promising therapies to move forward into humans. Current experimental models, however, do not accurately predict drug outcomes in humans (19, 20). Although *in vitro* systems, and carcinogen-induced, engraftment, and genetically-engineered mouse models are essential in bladder cancer research, they do not possess the collective features (cancer heterogeneity, molecular complexity, invasion, metastasis, immune cell response) that are crucial to predicting success or failure of emerging therapies in humans (19–23). With the resurgence of immunotherapy and the understanding that the immune system plays a major role in the outcomes of many types of therapies (16–18, 24–29), it is especially critical that animal models possess a level of immunocompetence similar to that in human cancer patients. There is compelling evidence that dogs with naturally-occurring InvUC possess these collective features and can serve as a highly relevant animal model for the human condition to complement other models (30–32). This review will summarize the similarities (and differences) between InvUC in dogs and humans, and discuss some of the settings in which the canine model could be most useful. Expanding the application of this canine InvUC model is expected to greatly improve the outlook for humans and dogs facing urinary bladder cancer.

## Clinical and Pathological Characteristics of Canine InvUC and Similarities and Differences Between InvUC in Dogs and Humans

### Frequency and Clinical Presentation of InvUC

Bladder cancer comprises ~1.5–2% of all naturally-occurring cancers in dogs, a rate similar to that reported in humans (1, 2, 30). With estimates that 4–6 million pet dogs develop cancer in the US each year, this equates to more than 60,000 cases of InvUC in dogs each year (31). It is acknowledged that many of these cases will go undiagnosed and untreated, but this still leaves large numbers of dogs diagnosed with InvUC who could participate in clinical trials.

As in humans, InvUC is typically a disease of older age dogs with the reported mean and median ages at diagnosis ranging from 9 to 11 years (30, 31). A minority of dogs develop the cancer at a younger age, i.e., as young as 4–6 years of age. The female to male ratio of dogs with InvUC has been reported to range from 1.71:1 to 1.95:1 (30). Interestingly, in dogs in high risk breeds, the female to male risk is less pronounced (30). The female gender predilection in dogs differs from that in humans in which males are more likely to be affected (15). The reasons for this difference between the species are not known. One possible reason relates to smoking in humans, a causative factor for up to 50% of human bladder cancer (15, 33, 34). Over several decades, smoking has been more prevalent in men than women (34). Men have also traditionally had more occupational exposures to chemicals (34). With a long latency period (up to 30–40 years) between carcinogen exposure and cancer development in humans, the differences in exposures between men and women decades ago can be reflected in current InvUC cases. Another factor to consider regarding the gender differences between bladder cancer in humans and dogs is that most dogs diagnosed with InvUC have been neutered, typically at a young age, and this could affect their bladder cancer risk (30). In fact, the risk of InvUC is ~2-fold higher in dogs who have been spayed or neutered than it is for intact dogs (30, 35). Interestingly, dogs who have been spayed or neutered also have a higher risk for other cancers (35–37). The reasons for this are not yet known, but the differences are likely to be important in gaining a better understanding of the processes leading to the development of InvUC and other cancers, and dog studies could be very informative.

The presenting clinical signs of InvUC are similar between dogs and humans, with hematuria being the most common change observed (30). Pain and urgency are not usually noted in the early stages of the cancer, but can emerge as the cancer progresses. A history of urinary tract infections is common in InvUC cases in humans and dogs. When cancer and infection are present concurrently, the clinical signs improve with antibiotic administration, but typically recur after the course of antibiotics is completed. As the cancer progresses, signs associated with metastases can emerge. Bone metastases, while uncommon, can lead to severe pain in both species.

### Pathological Features

**Figure 1:**
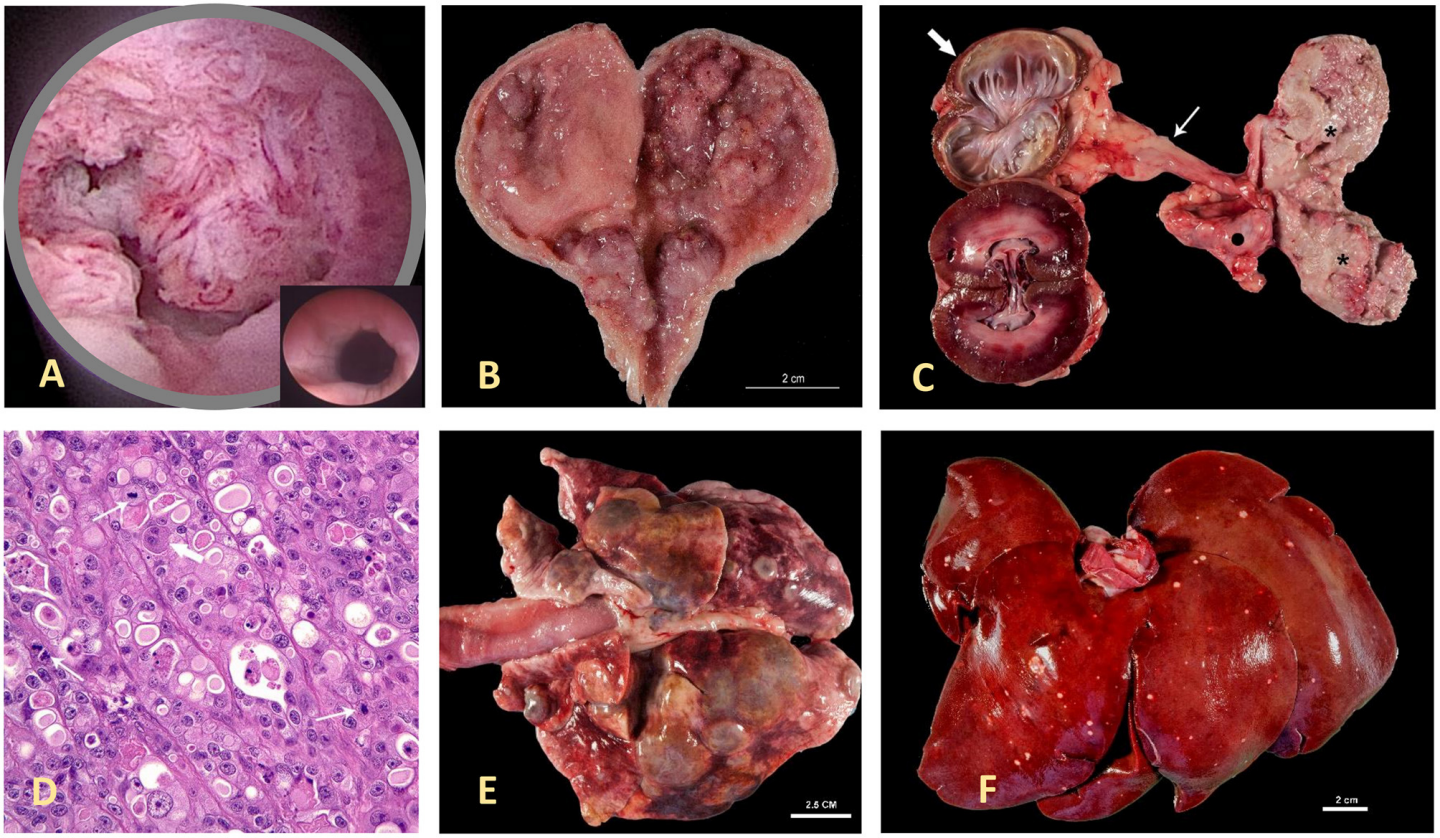
Canine invasive urothelial carcinoma (InvUC). Canine InvUC often produces papillary lesions extending into the lumen of the urethra (as seen in the cystoscopic image in **A**) and bladder (as seen on post mortem specimen in **B,C**), along with deep invasion into the bladder wall. For comparison, in **(A)**, the inset demonstrates the normal appearance of this region of the urinary tract in the absence of cancer. In (C), note the transmural growth in the entire bladder (please see the 2* on the right side of the panel), hydroureter (thin arrow), and hydronephrosis (thick arrow) caused by obstruction of the ureteral orifice by tumor growth in the bladder. An adjacent iliac lymph node (dark dot) is also infiltrated by this neoplasm. The photomicrograph in **(D)** (H&E 40X) is typical of high-grade InvUC. There is lack of normal cell maturation and marked nuclear atypia with some binucleated and multinucleated cells, and mitotic figures (arrows). Note the presence of cytoplasmic vacuoles within neoplastic cells, a common but not unique finding to InvUC. Canine InvUC is locally aggressive and metastasizes to distant sites in more than 50% of cases. Note metastases to the lung **(E)** and liver **(F)**. The gross appearance of metastases range from single to multiple nodules that can become confluent as observed in the lung **(E)**.

The diagnosis of InvUC in dogs and humans is made by histologic examination of tissue biopsies. In dogs, these tissues are collected by surgery, cystoscopy, or catheter biopsy (30). The vast majority of bladder cancer in dogs (>90% of cases) consists of intermediate- to high-grade InvUC, the focus of this review (Figure 1) (30). Great similarity is noted in the microscopic features between canine and human InvUC (30). It is interesting to note that superficial, low-grade bladder cancer is very uncommon in dogs. Another interesting difference between bladder cancer in dogs and humans is the location of the cancer within the bladder. The majority of InvUC in dogs is found in the trigone region of the bladder, and extension down the urethra is common (reported in >50% of cases) (Figure 1), whereas in humans there is a more balanced distribution of the cancer across different areas of the bladder (30). In male dogs, 29% of cases have been reported to have prostate involvement based on evidence from imaging studies or pathology (30). In humans, prostate involvement of the urothelial carcinoma has been found in cystoprostatecomy sections in 15–48% of cases across multiple studies, with higher rates of involvement noted with more detailed pathologic examination of the tissues (38). Interestingly, incidental prostatic adenocarcinoma has been found in 12–51% of cystoprostatectomy sections from men with InvUC (39). Primary urothelial carcinoma of the prostatic urethra and ducts (in the absence of bladder disease) is not uncommon in dogs, but is considered rare in humans (40). It is possible that the growing practice of neutering male pet dogs, especially at an early age, could reduce the risk of prostatic adenocarcinoma in dogs, while enhancing the development of urothelial carcinoma, although confirmation of this requires further study. Upper tract, i.e., renal pelvic, urothelial carcinoma is reported in 5–10% of humans (41). Similar lesions are found in dogs (especially at necropsy), but the frequency at which they occur has not yet been established.

Several classification and grading schemes have been published for canine urothelial neoplasms, particularly InvUC, and these have been summarized in a prior review (30). By growth pattern, InvUCs are divided into papillary (50%) or non-papillary (50%) tumors and infiltrating (≥90%) or non-infiltrating (≤10%) tumors. Although non-infiltrating tumors comprise the majority of human InvUCs (≥65% of cases), this form of bladder cancer is uncommon in dogs.

When surgical specimens are examined, there is typically sufficient tissue to assess growth pattern, depth of invasion, vascular invasion, etc. When tissue biopsies are obtained by cystoscopy, however, the size of the biopsy is often small, especially in dogs. Nuclear grade has been established as one of the few constant features that can be evaluated even in small tissue samples (30). In a retrospective study using nuclear grade as the only feature to assign grade, of 232 canine InvUCs (biopsies or postmortem specimens), two InvUCs were grade I; 67 InvUCs were grade II; and 163 InvUCs were grade III (30). Although this work utilized a three tier grading system, a four tier grading system is currently in use in dogs, reflecting that used in humans (42).

Histologic variants of urothelial carcinoma (also called urothelial carcinoma with divergent differentiation) have been increasingly reported in humans, and in some cases may have prognostic significance (43–45). Interestingly, the percentage of cells showing divergent differentiation does not appear to influence patient outcomes (44). Some of the urothelial carcinoma variants have been observed in dogs including plasmacytoid and rhabdoid types (46). While there have been very few reports of such variants to determine their clinical significance in dogs, in one study, canine InvUC with fibromyxoid stroma was associated with invasion of the muscle layer, suggesting a more aggressive behavior (47). Perhaps of more importance from a clinical perspective, will be to determine which genetic fingerprints within these variants can be exploited for targeted therapies.

### Local Invasion and Metastatic Behavior

InvUC in humans is characterized by locally aggressive cancer with growth into and often through the bladder wall, as well as distant metastases in ~50% of patients (15, 16). One of the reasons there is great enthusiasm for the canine InvUC model is the model replicates this local invasion and distant metastases of human InvUC, while these features are difficult to produce in experimental models. In dogs, nodal and distant metastases have been reported in ~16% of dogs at diagnosis and 50–60% of dogs at death (30). When applying World Health Organization (WHO) criteria for staging canine bladder tumors (Table 1) (48), 78% of dogs have been reported to have T2 tumors and 20% of dogs to have T3 tumors (30). There is a difference in the TNM staging system between dogs and humans, with T2 tumors in dogs including muscle invasive disease, whereas muscle invasive tumors in humans are typically classified as T3 or higher. Interestingly in a recent report of 65 dogs with InvUC that had whole body computed tomography (CT) performed at diagnosis, iliosacral lymphadenomegaly, sternal lymphadenomegaly, bone metastasis, and lung metastasis were suspected in 48, 18, 25, and 35% of the dogs, respectively (49). These rates of metastases appear higher than reported in other studies (30). It is possible that this group of dogs had later diagnoses when the cancer had become more advanced or that the CT imaging revealed more metastases than are typically observed with other imaging modalities.

**Table 1:** WHO TNM clinical staging system for canine bladder cancer (48).

**T—Primary tumor**	
Tis	Carcinoma *in situ*
T0	No evidence of a primary tumor
T1	Superficial papillary tumor
T2	Tumor invading the bladder wall, with induration
T3	Tumor invading neighboring organs (prostate, uterus, vagina, and pelvic canal)
**N—Regional lymph node (internal and external iliac lymph node)**	
N0	No regional lymph node involvement
N1	Regional lymph node involved
N2	Regional lymph node and juxtaregional lymph node involved
**M—Distant metastases**	
M0	No evidence of metastasis
M1	Distant metastasis present

To better characterize the distribution of InvUC metastases in dogs as the cancer progresses, necropsy findings were compiled from 137 dogs with InvUC evaluated at Purdue University (Table 2) (30). Of the 137 dogs, 92 dogs (67%) had metastasis to at least one site. Nodal metastases alone (in the absence of distant metastases) were found in 9% of dogs, distant metastases alone were found in 25% of dogs, and a combination of nodal and distant metastases were identified in 33% of dogs at the time of death (30). The frequency of metastasis and the sites involved were similar between dogs and humans (Table 2), with lung being the most common site of distant metastasis (50). In addition to visceral and nodal metastasis, InvUC also spreads to the abdominal wall through instruments and needles used in surgical and non-surgical procedures and naturally along ligaments that support the bladder (51). In this location, the cancer typically grows aggressively and is poorly responsive to medical therapy.

**Table 2:** Metastases identified in 137 dogs with invasive urothelial carcinoma undergoing necropsy at Purdue University (2005–2013) with comparison to published autopsy findings from 308 humans with urothelial carcinoma (30, 50).

**Location of metastases**	**Number of dogs with metastasis in that location (% of 137 dogs undergoing necropsy) (30)**	**Number of humans with metastases in that location (% of 308 humans undergoing autopsy) (50)**
Any metastases	92 (67%)	214 (69%)
Any nodal metastases	57 (42%)	180 (58%)
Regional nodes (abdominal, pelvic inguinal nodes)	40 (29%)^a^	158 (51%)
Thoracic nodes	17 (12%)^b^	80 (26%)
Other nodes	1 (1%)	8 (3%)
Any distant metastases	80 (58%)	147 (48%)
Lung	69 (50%)	96 (31%)
Bone	15 (11%)	71 (23%)
Liver	10 (7%)	103 (33%)
Kidney	10 (7%)^c^	30 (10%)
Adrenal gland	8 (6%)	28 (10%)
Skin	8 (6%)	4 (1.5%)
Spleen	6 (4%)	11 (3.6%)
Gastrointestinal	3 (2%)^d^	45 (15%)
Heart	5 (4%)	13 (4%)
Brain	2 (1.5%)	8 (2.5%)

a *Nodes included 32 iliac, sacral, and other “sub lumbar,” three inguinal, two mesenteric, two pancreatic, and one hypogastric node*.

b *Nodes included nine tracheobronchial, four sternal, three mediastinal, and one hilar node*.

c *It was not always possible to determine if the InvUC represented a second primary site in the kidney or a metastatic lesion*.

d *Tumor location included stomach in one dog, jejunum in one dog, and pancreas in one dog*.

Bone metastases are also important metastatic sites in dogs, as well as in humans. To assess the frequency of bone metastases in dogs, 188 dogs with InvUC undergoing necropsy were retrospectively studied (52). Of the 188 cases, 17 (9%) had histologically confirmed skeletal metastasis, mainly to the vertebrae. This was followed by a prospective study of 21 dogs with InvUC that underwent total body CT at the time of euthanasia followed by a standardized pathologic examination (52). In four dogs, skeletal lesions suspicious for bone metastases were detected on CT, and were confirmed to be InvUC metastases histologically in three (14%) dogs (52).

There was an additional interesting finding from the necropsy study of the 137 dogs with InvUC (30). Of the 137 dogs, 18 dogs (13%) had second primary tumors including hemangiosarcoma (*n* = 3), marginal zone lymphoma (*n* = 3), hepatocholangio-carcinoma (*n* = 2), follicular thyroid carcinoma (*n* = 2), B cell lymphoma (*n* = 2), adrenal adenocarcinoma (*n* = 1), meningioma (*n* = 1), nasal adenocarcinoma (*n* = 1), cutaneous squamous cell carcinoma (*n* = 1), oral melanoma (*n* = 1), pancreatic adenocarcinoma (*n* = 1), undifferentiated neuroendocrine tumor (*n* = 1), histiocytic sarcoma (*n* = 1), and splenic sarcoma (*n* = 1). Second primary tumors are also noted in humans with InvUC. In an autopsy study of 376 humans with InvUC, 74 patients (20%) had second primary tumors with other carcinomas being most common (53).

## Molecular Features in InvUC, and Similarities Between Dogs and Humans

### Molecular Subtypes

One of the compelling recent advances in InvUC is the identification of gene expression patterns that segregate human InvUC into molecular subtypes including basal, luminal, and others initially described in human breast cancer (13–17, 54, 55). The subtypes are important because there is strong evidence that cancer behavior and response to therapy differ between subtypes, and thus subtypes could emerge as parameters to use in individualizing cancer treatment (13–17).

To briefly summarize some of the findings regarding subtypes, basal subtype InvUCs are more prevalent in women than men, are associated with squamous features, and are enriched for *STAT3, TP63, KRT5/6A*, and *CD44*; and NFkB, c-Myc, and HIF signaling (13–17). Some basal InvUC also express epithelial-mesenchymal transition markers of claudin-low breast cancer (56). Basal InvUC is thought to be inherently more aggressive than other subtype tumors, and is associated with more advanced stage and metastatic disease at diagnosis, although basal InvUC can be responsive to chemotherapy and immunotherapy. Luminal subtype InvUC is associated with papillary histologic features and better clinical outcomes. The luminal tumors are enriched for *ER, TRIM24, FOXA1, GATA3, PPARG*, and activating *FGFR3* mutations (with good response to FGFR inhibitors) (14, 17, 57, 58).

**Figure 2:**
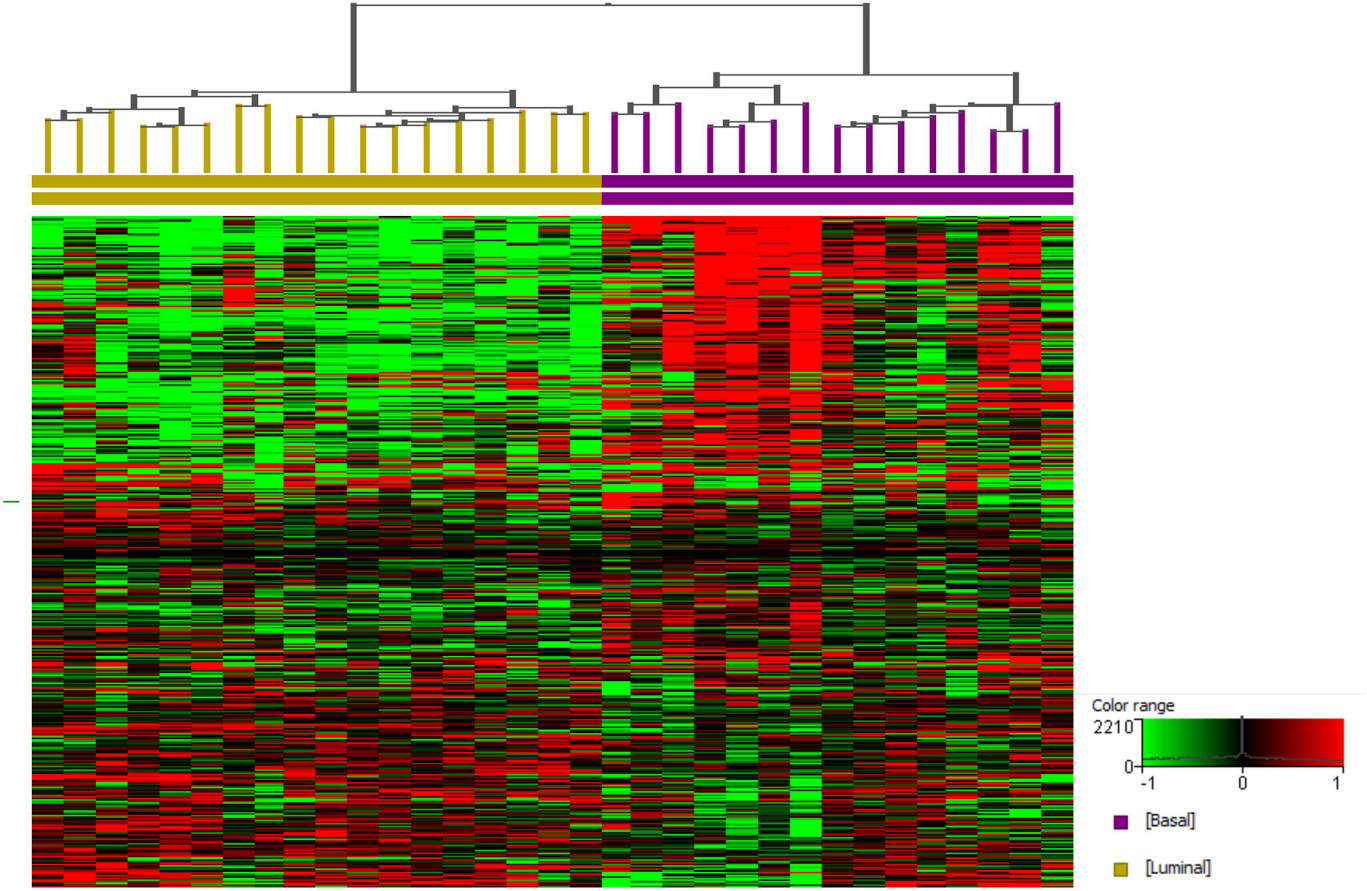
Basal and luminal subtypes in canine invasive urothelial carcinoma. RNA-seq data from canine InvUC (*n* = 33) and normal bladder mucosal samples (*n* = 4) were normalized using TMM and DESeq concurrently using Strand NGS (Strand, Bengaluru, India). Statistical analyses were conducted using edge R (on TMM normalized data) and DESeq2 (on DESeq normalized data) with *p* corr ≤ 0.05 and ≥2-fold change from each analysis. These two lists of differentially expressed genes were pooled as described previously (59). A prediction model reported earlier was employed to assign luminal and basal subtypes (59). Supervised hierarchical clustering was performed using genes that assign basal and luminal subtypes in human InvUC (17). Two distinct groups were identified as basal (*n* = 15) and luminal (*n* = 18).

It is clear that modeling drug effects across molecular subtypes is essential. Work by our group provides strong evidence that the molecular subtypes present in human InvUC are also present in canine InvUC. Briefly, RNA-seq data were analyzed from 29 canine InvUCs and normal control bladder tissues from four dogs with no evidence of bladder disease (59). In unsupervised clustering, the tumors clearly segregated into two groups. When the same data were analyzed using a panel of genes known to distinguish luminal from basal bladder tumors in humans, the two groups from the unsupervised clustering analyses were identified as luminal and basal subtype. This finding is depicted in Figure 2 in which additional cases have been added.

### Other Molecular Features

The molecular characterization of canine InvUC is still in the early stages, especially in regards to mutation signatures and epigenetic events. Some of the initial findings are summarized in Table 3 and in the following text.

**Table 3:** Early findings of molecular features in canine InvUC, and similarities and differences between canine and human InvUC.

**Molecular feature**	**Human InvUC**	**Canine InvUC**
Molecular subtypes	Luminal and basal are the main subtypes. Subgroupings within these occur (13–17)	Luminal and basal are the main subtypes. Early data indicating subgroupings requires confirmation in larger studies (59)
P53 pathway	p53 protein (presumed mutant) is detected by immunohistochemistry (IHC). Note: wildtype p53 protein is typically degraded faster than mutant p53, and thus mutant p53 is the form that is thought to be more commonly detected by IHC (15, 60)	p63 (homolog of p53) protein (presumed wildtype) is less abundant in InvUC than in normal bladder in IHC studies (61)
	Loss of function mutations in p53 occur in 50% of cases. Thus, the tumor suppressor pathway is inactivated (15)	P53 mutations are not well defined in canine InvUC. Enrichment has been noted in genes that negatively regulate the expression of p53 in microarray and RNA-seq analyses (59, 62)
	Loss of p53 function is often accompanied by loss of RB1 and amplification of MDM2 and CDKN2A (15)	RB1 expression is reduced in 5 of 8 canine InvUC cell lines (63). RB1 status has not yet been reported in clinical studies. MDM2 is overexpressed in InvUC in RNA-seq data. CDKN2B is overexpressed in RNA-seq data; CDKN2A has not been reported (59)
PTEN/PI3K/AKT/mTOR pathway	Aberrant pathway activation occurs in 40% of human cases (13)	Overexpression of some genes in the pathway have been observed, but further study is needed to better characterize the pathway in dogs and to define similarities and differences between dogs and humans (59)
RTK/RAS pathway	FGFR3 mutations occur in 20% of cases (13)	FGFR3 mutations have not yet been defined
	BRAF mutations are rare (13)	BRAF^V600E^ mutations are common (~80% of cases) (64, 65)
EGFR	EGFR is overexpressed in 75% of high grade tumors detected by IHC (66, 67)	EGFR is overexpressed in 73% of cases as detected by IHC (62)
Cox-2	Cox-2 is overexpressed in >80% of cases as detected by IHC	Cox-2 is overexpressed in >80% of cases as detected by IHC and RNA-seq analyses (30)

Several important molecular features of InvUC were identified in the 2014 Cancer Genome Atlas Research Network (TCGA) comprehensive molecular characterization of human urothelial carcinomas (54). This study provided insight into the molecular pathogenesis of human InvUC, and identified potential treatment targets (54). Genomic alternations involving PI3K/AKT/mTOR, CDKN2A/CDK4/CCND1, and RTK/RAS pathways were noted, and thus receptor tyrosine kinases such as EGFR, ERBB2 (Her-2), ERBB3, and FGFR3 were identified as potential targets for therapy (54). Multiple sequencing studies of InvUC in dogs have been reported, some with cross species analyses, and many similarities across the species have been identified (59, 62, 68–71).

Overexpression of epidermal growth factor receptor (EGFR) has been reported in 73% of canine InvUC, which is comparable to that found in humans (62, 66, 67, 72). Inhibitors of EGFR family proteins have been evaluated in multiple human bladder cancer trials with varying success (73–75). EGFR inhibitors appear to be most useful in patients that are chemotherapy naïve and that have cancer overexpressing EGFR or ERBB2 (76). Prior treatment with chemotherapy can result in resistance to EGFR inhibitors, although the mechanisms of this are not well-defined. Canine studies could lead to a better understanding of the mechanisms of resistance, and to better identify subsets of patients who could benefit from EGFR inhibitor therapy.

One approach under investigation is to target EGFR with a photoimmunotherapy conjugate (Can225-IR700 conjugate), an approach which has shown promise in canine InvUC cell lines *in vitro* and in rodent models (77). The Can225-IR700 conjugate was shown to bind specifically and cause killing of EGFR expressing cells *in vitro*. In mice there was accumulation of the Can225-IR700 conjugate in the tumors with high tumor-to-background ratio, and tumor growth was significantly inhibited by near infrared photoimmunotherapy application (77). Using a different approach to exploit EGFR expression, a study of an EGFR-targeted toxin has recently been reported with antitumor activity observed in dogs with InvUC (78). These canine studies are being conducted to determine promising approaches to take into human clinical trials.

In considering a different molecular target, HER-2 (EGFR2/ERBB2/NEU) has been found to be significantly overexpressed in canine InvUC samples when compared to non-neoplastic urothelium (79), as is the case in human InvUC (80, 81). In two immunohistochemical studies HER-2 immunoreactivity was noted in 13 of 23 (56%) and in 14 of 23 (60.9%) cases of canine InvUC, respectively (79, 82).

The *p53* tumor suppressor gene product has an important role in differentiation of the urothelium (60). Loss of p53 expression has been noted in human InvUC, and has been associated with lymph node metastasis, advanced TNM stage, and shorter survival times (60, 83). Similar to the reports in humans, p63 expression, a homolog of p53, has been reported to be significantly lower in dogs with InvUC, compared to dogs with polypoid cystitis and normal urothelium (61). Expression of p53, the p53 inducible gene 14-3-3σ protein, and vimentin have been documented in canine InvUC *in vitro* and *in vivo* (63, 84). Expression of vimentin in human InvUC has been associated with epithelial-mesenchymal transition, cancer progression, and metastasis (85). The 14-3-3σ protein, which is expressed in human and canine bladder cancer, has also been linked to tumorigenesis (86, 87).

Mutations in several other genes implicated in the development and progression of InvUC and other cancers in humans have been identified in canine InvUC (64, 69). Examples include *CDKN2B, PIK3CA, BRCA2, NFkB, ARHGEF4, XPA, NCOA4, MDC1, UBR5, RB1CC1, RPS6, CIITA, MITF*, and *WT1* (16, 54, 64, 69, 88–93). It is anticipated that other shared molecular targets will be found. In early microarray analysis, more than 450 genes were identified that were differentially expressed between InvUC and normal bladder and that were shared between dogs and humans (*P* < 0.05; 2FC) (62, 94). In one report involving RNA-seq analysis, 1,589 genes were identified that were differentially expressed between normal bladder and bladder cancer in dogs and in humans (69).

Along with the notable similarities between canine and human InvUC, there is one intriguing difference. The majority (67–85%) of canine InvUCs harbor a *BRAF*^V595E^ mutation, which is homologous to the *BRAF*^V600E^ mutation in humans (64, 65). This mutation is considered a driver mutation of 8% of all human cancer across cancer types, and is especially common in human metastatic melanoma (64, 95). This mutation leads to constitutive activation of the MAPK pathway. While *BRAF* mutations are common in certain forms of human cancer, these mutations are rare in human InvUC. Other mutations within the MAPK pathway, however, occur in ~30% of human InvUC cases (54). It is intriguing that even though *BRAF* mutations are common in canine InvUC and that different molecular drivers are more common in human InvUC, the cancer in both species converges into a disease possessing the same molecular subtypes.

In addition to traditional methods to assess molecular features in cancer, canine InvUC has been used as a test case for other methodologies. For example, the methods for desorption electrospray ionization (DESI), an ambient ionization mass spectrometry approach, were developed using canine tissues (96). In DESI analyses, lipid patterns were identified that distinguish InvUC from normal urothelium in the canine tissues. In a follow up study, similar lipid patterns were found in human bladder cancer tissues (96, 97). Recently a new ambient ionization MS approach, touch spray MS (TS-MS), has been tested in canine tissues, and this technique rapidly identified lipid patterns that distinguished InvUC from normal tissues (98). This technique is especially intriguing because optimization of this form of MS could lead to a point-of-care instrument for use in the operating room or cystoscopy suite.

## Similarities in Treatment Response Between Dogs and Humans With InvUC

### Standard Treatments for InvUC in Humans and Dogs

Some of the key features concerning the treatment of InvUC in humans and dogs are summarized in Table 4. In humans, the standard treatment for bladder-confined InvUC is cystectomy, usually combined with neoadjuvant chemotherapy (8, 9). In half of patients, distant metastases emerge over the next 1–2 years and sometimes later, and the metastatic disease is treated with chemotherapy or immunotherapy (4, 10, 12). For patients who are not eligible for cystectomy, bladder sparing therapies combining radiation therapy and chemotherapy have been defined (99).

**Table 4:** Treatment options for invasive urinary bladder cancer in humans and dogs.

**Type of Therapy**	**Human InvUC**	**Canine InvUC**
Cystectomy	Cystectomy is the frontline treatment of choice in eligible patients with bladder-confined cancer. It is typically combined with neoadjuvant chemotherapy (8, 9)	Cystectomy is not usually performed in pet dogs due to the morbidity and cost of the procedure, and frequent extension of the cancer down the urethra which could preclude surgical cure (30–32)
Radiotherapy	Radiotherapy is used in trimodal therapies (maximum transurethral resection, radiotherapy, chemotherapy) in bladder sparing protocols. This is typically reserved for patients who are not eligible for or choose to forego cystectomy (99)	Studies to determine the efficacy of radiotherapy in dogs are limited. Trimodal therapy has not been investigated in dogs (100–102)
Chemotherapy	Chemotherapy is most often used in the neoadjuvant setting and in the treatment of emergent metastasis. Chemotherapy protocols can include: MVAC (methotrexate, vinblastine, doxorubicin, cisplatin), or in recent years less toxic combinations such as cisplatin-gemcitabine or carboplatin-taxol (103, 104)	Since cystectomy is rarely performed in dogs, chemotherapy is used to treat the primary cancer in the urinary tract, as well as to treat metastasis. Chemotherapy drugs with activity in dogs include: cisplatin, carboplatin, vinblastine, mitoxantrone, and others. Cisplatin is considered one of the most active agents in humans and dogs, but is rarely used in dogs due to consistent renal toxicity (10, 30)
Cyclooxygenase (Cox) inhibitors	Cox inhibitors are not routinely used as anticancer agents in human bladder cancer. In humans, Cox inhibitors induce biological changes in tumor tissues similar to those noted in canine bladder cancer (105, 106)	Cox inhibitors are a mainstay of canine bladder cancer treatment. These drugs are appealing because of the antitumor effects (single agent remission rate 20%, stable disease rate 55–60%), oral delivery, relatively low cost and risk of side effects, and positive benefits on quality of life. Cox inhibitors are also used to improve remission rates with chemotherapy, e.g., doubling the remission rate with cisplatin and vinblastine (30, 107–112)
Immunotherapy	Immune checkpoint inhibitors approved for use in humans include those targeting PD-L1 (atezolizumab, durvalumab, avelumab) and those targeting PD-1 (pembrolizumab, nivolumab) (12, 113–120)	Immune checkpoint inhibitors are not yet available for use in dogs
Targeted agents	An FGFR inhibitor (Erdafitinib) is approved for use in human bladder cancer	FGFR mutations are less common in canine bladder cancer, and agents targeting FGFR have not been tested in dogs. Targeted therapies tested in dogs include an EGF-toxin conjugate, and folate targeted therapies (78, 121, 122)

The treatment of InvUC in dogs can include surgery, radiation therapy, chemotherapy and other drugs, or combinations of these, although surgery and radiation therapy are used less often than drug therapy in dogs (30–32). Complete cystectomy is not typically performed in pet dogs because of the frequent extension of cancer beyond the bladder (urethra, prostate, other organs), the morbidity of the procedure, and the expense involved (30–32). Most InvUC lesions in dogs are not in a location where complete surgical excision is possible. Early reports of radiation therapy in dogs with InvUC were discouraging because of the side effects (100), although newer radiation therapy approaches have been much better tolerated, allowing further study (101, 102). Currently, drugs are the mainstay for treatment of InvUC in dogs (30). Pet owners insist that the drug protocols be well-tolerated; anything beyond mild side effects is not considered acceptable. This is not unreasonable, and low adverse event profiles are also desirable for humans. Although InvUC is not usually curable in dogs with current therapies, the disease can be controlled in 80% or more of dogs, and the dogs can enjoy many months to beyond a year, with a minority of dogs living more than 3 years with good quality of life (30). It should be noted that since the bladder is not removed, dogs can be used to study treatments of organ-confined disease, metastases, or both. It is recognized that having the primary tumor intact will lead to continued emergence of cells with metastatic potential.

### Chemotherapy Responses

The response to chemotherapy for InvUC is similar between dogs and humans. Platinum agents are considered to be the most active agents in both species (10, 30, 103, 104). Cisplatin-based combination chemotherapy protocols are not often used in dogs because of side effects considered unacceptable in dogs, although a comparison of single-agent activity between dogs and humans is possible. The remission rate with single-agent cisplatin has been reported to be 12–20% in dogs and 17–34% in humans (30, 104). Carboplatin has activity in both species, although it is considered less active than cisplatin (30, 104, 123). The previous standard protocol for InvUC treatment in humans was methotrexate, vinblastine, doxorubicin, and cisplatin (104). Although this protocol was considered too toxic for acceptable use in pet dogs, an important component of the protocol, vinblastine, has been evaluated in dogs with remission and stable disease rates of 36 and 50%, respectively (124). Vinblastine has single-agent activity in humans and contributes to combination therapy protocols (125, 126). Gemcitabine is also considered an active drug in both species (127, 128).

## Enthusiasm for Canine Clinical Trials in InvUC, and Examples of Translational Studies

### Canine Clinical Trials, a Win-Win-Win Scenario

Treatment studies in dogs are expected to be a win–win–win scenario (30). The individual dog receives treatment that is expected to help them and that often provides hope when other treatments are not effective or not feasible. The study results are expected to help other dogs and ultimately help humans with InvUC. The subsidized cost for treatment in many of the trials allows some pet owners to pursue treatment for their dog even if they cannot afford any other therapies. For all of these reasons, in the Purdue University Veterinary Teaching Hospital, more than 90% of owners of dogs with InvUC elect to enroll their pet in a clinical trial. Parallel mechanism studies are feasible in dogs with samples of blood, urine, and, in some cases, tumor tissues collected by cystoscopy available before, during, and after therapy. Most pet owners will also allow a necropsy of the dog when it dies or is euthanized (because of declining quality of life due to cancer progression or other conditions). This provides crucial information on the disease process and response to therapy, and the opportunity to bank tissue samples for future studies. Although most treatments tested in dogs have been systemic therapies, dog studies can also be used to evaluate intravesical therapy (129).

### Evaluation of Emerging Targeted Therapies in Dogs With InvUC

There are published examples of studies of targeted therapies in dogs with InvUC of translational value (121, 122, 130). One example is a canine clinical study performed to determine the expression of high affinity folate receptors (folate receptor alpha) in InvUC and the safety and efficacy of folate-targeted therapy (121). Briefly, folate receptor alpha expression was detected in 78% of canine InvUC tissues, and folate uptake *in vivo* was confirmed by scintigraphy. An escalating dose of folate-targeted vinblastine (EC0905) was administered to pet dogs with biopsy-confirmed folate receptor-positive InvUC. The maximum tolerated dose was determined, with neutropenia and gastrointestinal upset being dose limiting toxicities. The drug was well-tolerated at the maximum tolerated dose, and good antitumor activity was observed (121). Folate receptor expression was identified in human InvUC (121), and further work is ongoing to define the percentage of cases with folate receptor expression. Although the folate-vinblastine conjugate had good antitumor activity in dogs with InvUC, the duration of remission was limited in many cases. Thus, a follow up study was performed in dogs using a different folate conjugate, folate-tubulysin (EC0531) (122). Unlike vinblastine, tubulysin is not a substrate for the P-glycoprotein drug efflux pump, and therefore, longer remission times were anticipated (122). In the EC0531 study, the maximum tolerated dose was defined, and again neutropenia and gastrointestinal toxicity were observed at higher doses, as were observed with folate-vinblastine treatment. Of 28 dogs treated, three dogs had partial remission and 17 dogs had stable disease (122). The progression free interval appeared longer than that noted in dogs treated with folate-vinblastine, although a head-to-head comparison would be required to confirm this. Unlike human neutrophils, canine neutrophils were found to express folate receptors, which contributes to the neutropenia at higher doses of folate-targeted therapies in dogs (122). This suggest that humans may tolerate higher, potentially more effective, doses of folate-targeted therapies.

### Evaluation of Cox Inhibitors in InvUC

An intriguing discovery was made in dogs with InvUC and other types of cancer which is expected to translate into benefit in humans. Briefly, non-selective cyclooxygenase inhibitors (Cox inhibitors, i.e., non-steroidal anti-inflammatory drugs) have had unexpected antitumor effects in dogs with cancer (107). The interest in Cox inhibitors in dogs with cancer stemmed from the observation of dramatic remission of a poorly differentiated sarcoma of the thoracic wall in one dog and of complete remission of advanced metastatic carcinoma of unknown primary in another dog who were receiving the non-selective Cox inhibitor, piroxicam, but no other drugs (107). These initial observations made more than three decades ago subsequently led to phase I, II, and III clinical trials of Cox inhibitors in dogs with InvUC which confirmed the antitumor effects and safety of the drugs (30, 107–110). In 76 dogs with InvUC treated with single-agent piroxicam, tumor responses included two (3%) complete remission (complete resolution of all clinical evidence of cancer), 14 (18%) partial remission (≥50% reduction in tumor volume and no new tumor lesions), 45 (59%) stable disease (<50% change in tumor volume and no new lesions), and 15 (20%) progressive disease (≥50% increase in tumor volume or the development of new tumor lesions) (30).

In addition to the antitumor effects of single agent Cox inhibitor treatment, these drugs also enhance the activity of chemotherapy (109–111). In dogs, Cox inhibitors have enhanced the activity of cisplatin in multiple studies including randomized trials (109, 111, 112). The remission rate with cisplatin alone was <20%, while the remission rate with cisplatin combined with the Cox inhibitor ranged from 50–70% across randomized trials (109, 111, 112). Similarly, in another randomized trial in dogs with InvUC, the remission rate was significantly higher in dogs receiving vinblastine combined with piroxicam (58%) than in dogs receiving vinblastine alone (23%) (110).

The findings from the Cox inhibitor studies in dogs have been translated into humans with InvUC (105). Intriguingly, the biological effects associated with Cox inhibitor-induced remission in dogs (e.g., induction of apoptosis) were found to occur to the same degree in humans with InvUC receiving the Cox-2 inhibitor, celecoxib prescribed between initial diagnosis and cystectomy (105, 106). Cox inhibitors have also reduced the recurrence of superficial bladder tumors in humans in some, but not all studies (131, 132).

Proposed mechanisms of the antitumor effects of Cox inhibitors have included antiangiogenic effects, immunologic effects, modulation of cancer stem cells, and direct induction of apoptosis (105, 106, 133, 134). There are growing numbers of studies of the immunologic effects. Cox-2 is upregulated in canine and human InvUC (135, 136). Cox and the Cox product PGE2 in tumor-associated macrophages and tumors, have been reported to decrease the activation and proliferation of T cells (CD4+, CD8+), increase release of IDO1, reduce the function of NK cells, cause a shift from Th1 to Th2 response, increase the infiltration of regulatory cells into the tumor and release of immunosuppressive cytokines, decrease immunostimulatory cytokines, and to drive negative DAMPs (damage-associated molecular patterns) (137–141). Cox blockade (via knockdown or drugs) has been shown to reverse all of these effects (137–141). Of special current interest, aspirin enhanced the effects of immune checkpoint inhibitor treatment of melanoma and colon tumors in mice (141). These and other reports prompted our group to re-examine H&E slides from patients with InvUC in the celecoxib trial (105). Interestingly, the number of tumor-infiltrating lymphocytes (TILs) increased multifold in 73% of cases receiving celecoxib, compared to 38% of control cases (Dhawan and Knapp, unpublished data). Clearly, further studies of the effects of Cox inhibitors in InvUC treatment are indicated, and dogs offer an ideal animal model for this work.

## Growing Role for the Dog Model to Drive Advances in Emerging Immunotherapies for InvUC

### Emerging Role and Need for Advances in Immunotherapy

The medical community has seen an unprecedented resurgence in immunotherapy, as impressive remissions have been documented in patients with advanced chemotherapy-refractory cancer (12, 24, 25, 113–120). There is clear promise for immunotherapies, yet a crucial need to improve the effectiveness of these agents. This has heightened the demand for relevant immunocompetent animal models of cancer that can predict the outcomes (efficacy, toxicity) of immunotherapies (alone and when combined with other agents) when given to humans.

Among emerging immunotherapies, there is particularly high interest in immune checkpoint inhibitors (12, 24, 25, 113–120). Immune checkpoints, including PD-L1, PD-1, CTLA-4, B7x, and others, are critical regulatory components of the immune system that are essential for maintaining self-tolerance (24, 113). Immune checkpoints also modulate the amplitude and length of physiological immune responses in peripheral tissues in order to minimize collateral tissue damage. Many types of cancer, however, exploit these immune checkpoints to evade immune attack especially by T cells specific for tumor antigens (24, 113). Cancer cells upregulate PD-L1 (and other immune checkpoints) in response to oncogenic signals or endogenous antitumor immune responses. The binding of PD-L1 to PD-1 on activated T cells causes cell anergy or death (24, 113). PD-L1 is also expressed by antigen presenting cells, natural killer cells, and T cells, and can interfere with the function of these cells.

The finding of dramatic durable complete remissions in heavily pre-treated patients in multiple studies provides compelling evidence that immune checkpoint inhibitors can drive new success in treating InvUC and other cancers (25, 113–120). Much more work must be done, however, before immune checkpoint inhibitors reach their potential in saving the lives of cancer patients. Although impressive remissions are seen in patients with advanced cancer, only a minority of patients (~20%) have this level of benefit (25, 113, 115–120). In addition, immune checkpoint inhibitors can unleash a plethora of autoimmune processes, and special attention must be paid to monitoring and treating these “toxicities” (120). Studies of biomarkers to predict immune checkpoint inhibitor activity have had conflicting results, indicating the need for continued study (25, 113, 114, 142–145).

The limited rate of remission with immune checkpoint inhibitors and the absence of clear biomarkers of response are not surprising because the immune system can fail at multiple points in attacking the cancer (24, 26, 114). Causes of immune failure can include low antigenicity (e.g., lack of antigens, MHC downregulation), deficient adjuvanticity (e.g., lack of DAMPs) to signal the immune system, ineffective T cell trafficking, immunosuppressive cells and cytokines, exhausted T cells, and deficient numbers or function of immune effector cells in general (24, 146, 147). It is expected that combining drugs that positively affect different parts of the immune system will substantially increase the success rate of immune checkpoint inhibitors (26–29). This again highlights the need for relevant animal models to help select the most promising approaches to take into human trials.

There are multiple reports of the expression of immune checkpoints in InvUC and other cancers in dogs (148–157). In addition to the well-known checkpoints PD-1, PD-L1, CTLA-4, other checkpoints have been identified in canine InvUC (59, 157). B7x (B7-H4/B7S1/VTCN1), for example, is an inhibitory immune checkpoint molecule and is considered a potential therapeutic target because of its immunosuppressive effects and well-known expression in cancers (157). The expression of B7x in canine InvUC has recently been reported (157). In RNA-seq analysis, a 5–7-fold increase in the expression of B7x in canine InvUC was noted compared to the expression in the normal bladder. B7x protein expression was confirmed by immunohistochemistry (IHC) with medium to high expression in 18 of 50 (20%) canine InvUC samples studied (157). For comparison, TCGA and Genotype-Tissue Expression (GTEx) data sets were used to examine B7x expression in 599 human urothelial carcinomas. B7x expression was significantly (*p* = 0.02) associated with worse overall survival in humans (157).

The scientific community is eagerly awaiting the availability of immune checkpoint inhibitors for studies in dogs. Human monoclonal antibodies that target immune checkpoints have not yet been shown to bind and functionally disrupt canine checkpoints. In addition, neutralizing antibodies would form in dogs in response to the administration of human antibodies, i.e., foreign protein, making the antibody treatment ineffective and potentially leading to allergic and anaphylactic reactions in the dogs.

There are reports of canine PD-L1 (cPD-L1) antibodies developed by academic laboratories (155, 156). A canine chimeric PD-L1 antibody has been administered to nine dogs with oral melanoma or soft tissue sarcomas (155). Although tumor regression was observed in two dogs, the extent of the cPD-L1 inhibitor's activity is not known because the dogs were allowed to receive concurrent non-steroidal anti-inflammatory drugs that have been documented to have antitumor effects in those cancers in dogs and in canine xenograft models (107, 158, 159). The lack of immune-mediated toxicity (which is common in humans) in the dogs also calls the drug's activity into question.

As immune checkpoint inhibitors become available for dogs, studies to evaluate the antitumor effects, determine mechanisms of response and resistance, test potential combination therapies, and develop strategies to minimize adverse events will be high priorities. It is likely that dogs will develop adverse events similar to the autoimmune-related adverse events in humans because dogs naturally develop immune mediated diseases such as hemolytic anemia, thrombocytopenia, myasthenia gravis, polyarthritis, inflammatory bowel disease, and others (160). The adverse events are expected to be manageable in dogs, just as they are in humans. Dog studies of the antitumor effects, safety, and mechanisms of response and resistance to immune checkpoint inhibitors are anticipated to be key to advancing these therapies in humans.

Next to immune checkpoint inhibitors, chimeric antigen receptor T cells (CAR T cells) which are T lymphocytes engineered to express a specific chimeric antigen receptor, are gaining the most attention in immunotherapy, and are perhaps showing more promise than other current immunotherapy strategies (161). CAR T cell therapy has been successfully delivered to dogs with naturally-occurring lymphoma (162). Briefly, autologous RNA-transfected CAR T cells were generated, expanded, and administered to pet dogs with relapsed B cell lymphoma. The treatment was well-tolerated and resulted in reduction of CD20+ B cells in target lymph nodes. The results from this proof-of-concept study validate further evaluation of CAR T cell therapy in dogs, and the opportunity to fill the gap between mouse models and translation into humans (162).

### Monitoring the Immune Response in Dogs With InvUC

Although the fairly extensive “tool kit” available to assess immune cells and the activity of the immune system in humans is much more limited in dogs, methods do exist to analyze immune cells and cytokines in circulation and in the tumor masses. A few of those used to study the immune infiltrates in the tumor will be highlighted in this review.

**Figure 3:**
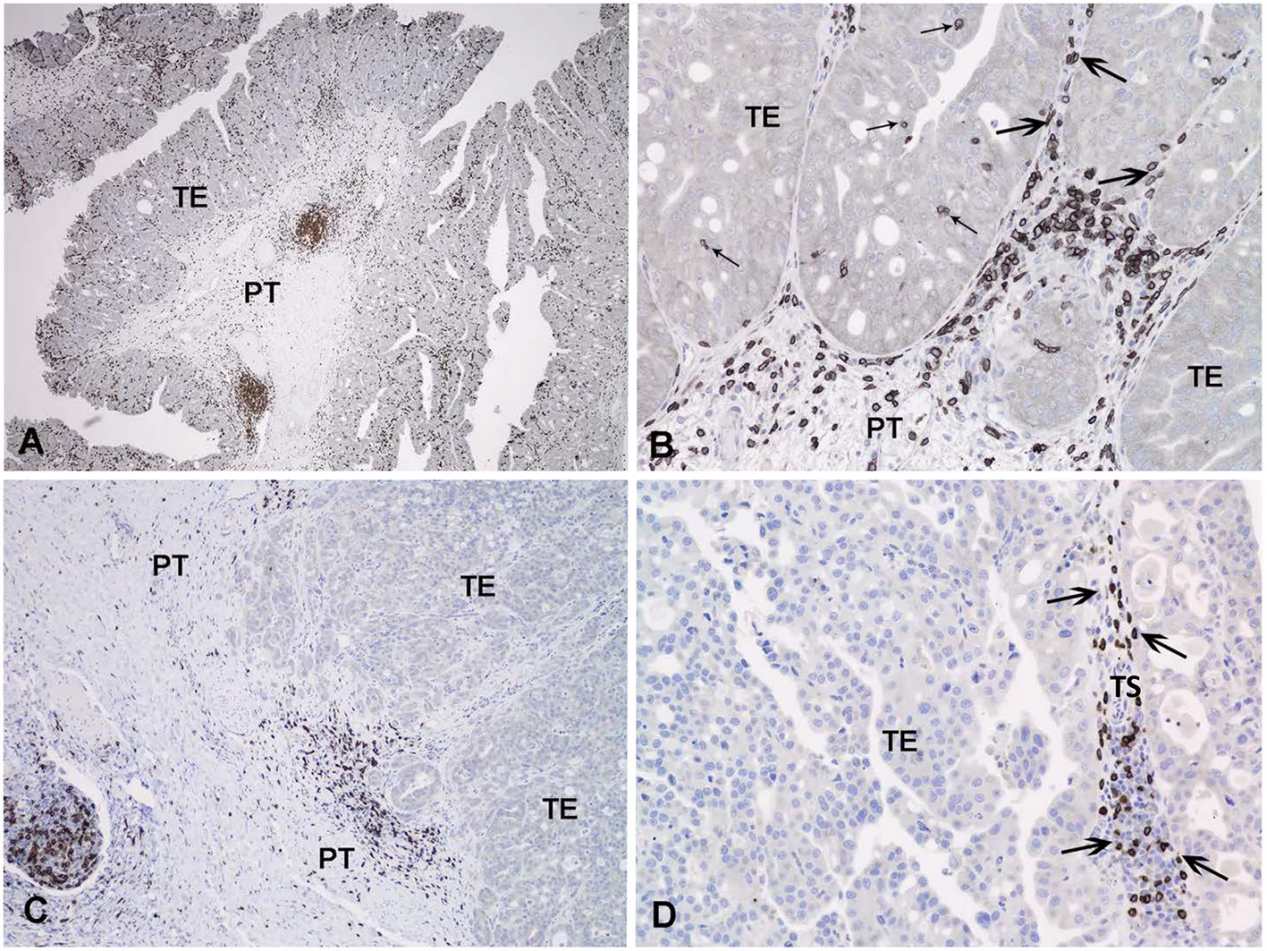
Immunohistochemical detection of T lymphocytes with an antibody to CD3 in canine invasive urothelial carcinoma. In **(A)** all areas examined (intraepithelial, tumor stroma, and peritumoral) contain CD3 positive cells. In **(B)** a detail of the triphasic pattern of CD3 expression is noted. In **(C)** only the peritumoral lymphoid infiltrate expresses CD3 in this tumor. In **(D)** the tumoral stroma contains numerous CD3 positive lymphocytes, but the tumor epithelium is negative. TE, tumoral epithelium; TS, tumoral stroma; PT, peritumoral stroma; Small arrow, intraepithelial T-lymphocytes; Large arrow, tumoral stroma T lymphocytes.

Immune cells infiltrating the tumor can be visualized with IHC (163–166). While all of the markers for various immune cells in humans are not available for dogs, CD3 IHC is a popular approach to assess tumor infiltrating lymphocytes (TILs) in canine cancer, including application to formalin fixed tissues (166). IHC protocols have also been described to detect regulatory T cells in canine tumors (167, 168). More specific immune cells can be detected in frozen sections of canine tumors (169). Using IHC, the pattern of TILs in human InvUC have been classified in some studies as: (1) immune desert (no or very few TILs observed), (2) immune excluded (TILs on the periphery of the tumor mass but no TILs within the mass itself), or (3) immune infiltrated (TILs in the mass), with further distinctions made for the presence of TILs in the stroma in and around the tumor or between tumor cells in the tumor mass (163–165). An effective immune attack is expected to require TILs within the tumor mass, and there is great interest in developing strategies to convert the immune desert or immune excluded state to an immune infiltrated state. It is therefore important to note that these same patterns of TILs have been observed in canine InvUC (Figure 3).

**Figure 4:**
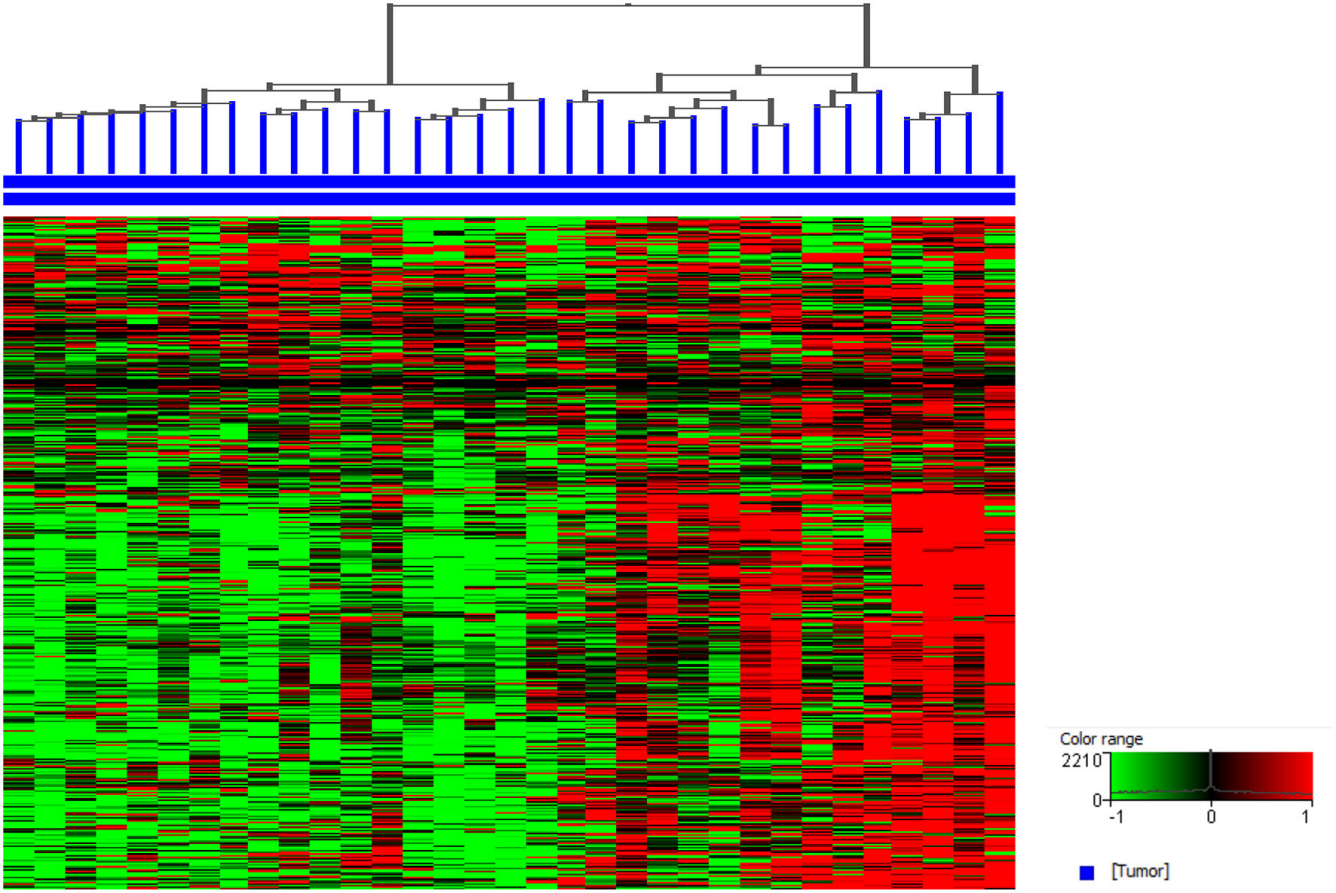
Canine invasive urothelial carcinoma (InvUC) samples display gene expression patterns classifying the tumors as immune infiltrated (immune “hot”) or non-immune infiltrated (immune “cold”). A list of immune signature genes known to be upregulated in T-cell inflamed human InvUC samples were used (170) to visualize the immune patterns that exist in canine InvUC. Normalized intensity values were used for supervised hierarchical clustering using Euclidean distance metrics and Ward's linkage algorithm as a distance metric. Note the predominantly high expression of immune genes in the right cluster of the canine InvUC samples (*n* = 15, 45%) classifying them as immune “hot” (immune infiltrated).

**Figure 5:**
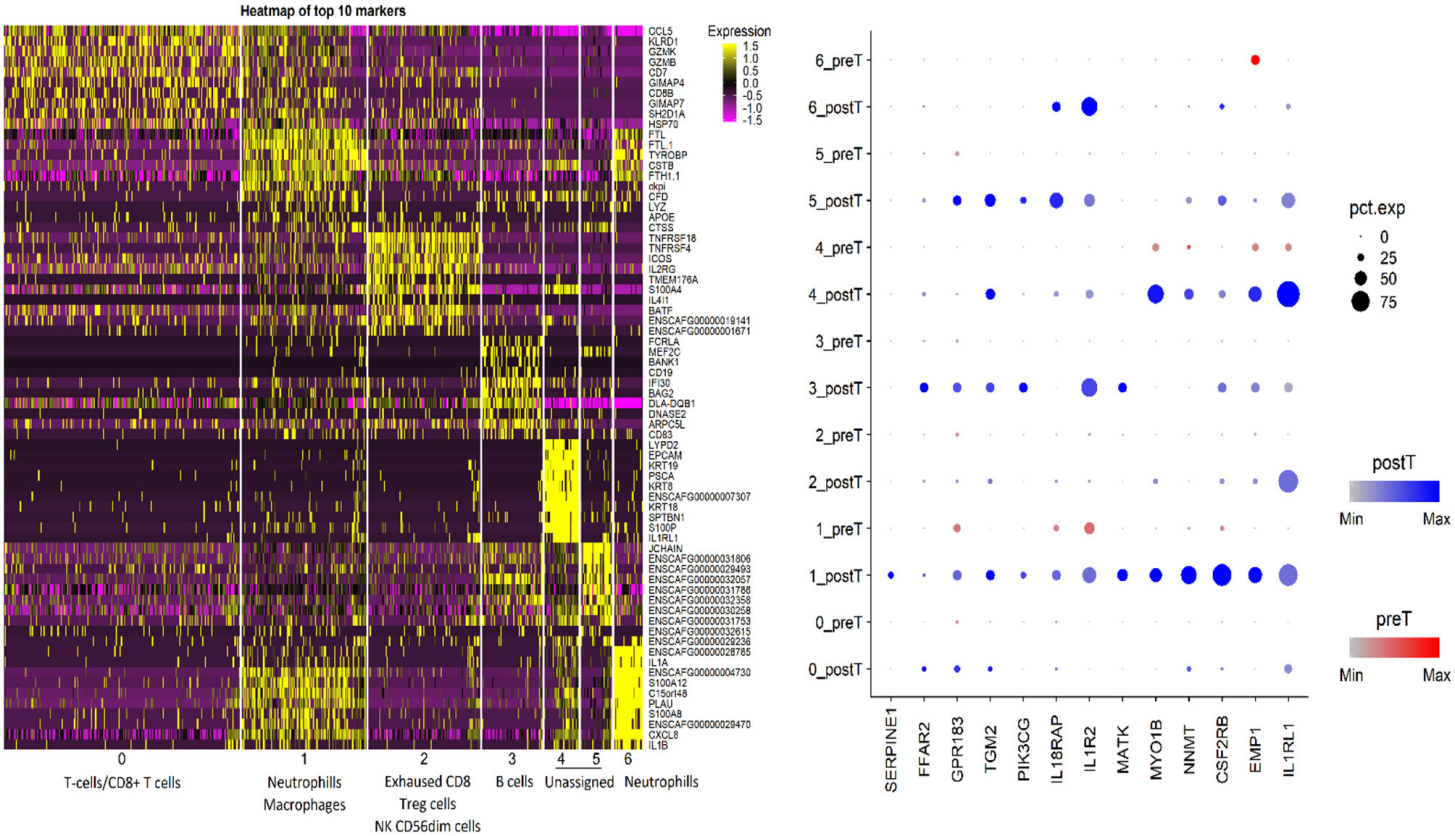
Single cell RNA-seq analysis of canine invasive urothelial carcinoma. Unsupervised clustering of the canine InvUC sample was performed (Seurat package, Satija Lab). The cells segregated into seven different clusters. Putative cell type assignment was based on marker gene expression and abundance within the cluster. The gene expression heatmap focused on the CD45+ cells (immune cells) shows the cell clusters with putative immune cell type assignments on x-axis and top 10 marker genes in each cluster on Y-axis. The split dotplot on right shows the intensity (dot color) and percentage of cells expressing (dot size) 13 marker genes (x axis) analyzed in InvUC tissue across clusters before and after treatment (y axis). This type of data can be used to study mechanisms and generate new hypotheses. For example, GPR183 which increases in cluster 1 cells, is known for its role in lymphoid organ development and positioning of activated CD4 T cells in lymphoid follicles, but its role in the immune state of InvUC has not been elucidated (174).

Sequencing studies have also been used to characterize the immune state in InvUC tissues (13, 16, 54, 59, 170–173). Whole exome sequencing analyses can be used to determine tumor mutation burden and neoantigen load, factors that are thought to influence the immune attack on the cancer (171–173). Patterns in RNA-seq data have been defined to classify tumors broadly as “immune hot” (immune infiltrated) or “immune cold” (non-infiltrated), with mixed patterns also present (13, 16, 163, 170). The immune hot tumors are expected to be primed to respond well to immunotherapy and other therapies, whereas the immune cold tumors are thought to be largely incapable of responding to immunotherapy. Similar RNA-seq analyses have been used to demonstrate an immune hot vs. cold state in canine InvUC (Figure 4) (59). This demonstrates that the canine InvUC model can be used to develop and test strategies to convert immune cold tumors to immune hot tumors in order to sensitize the tumor to immunotherapy. There are also intriguing initial findings from single cell RNA-seq analyses of canine InvUC (Figure 5) (174, 175). When performing single cell RNA-seq on InvUC tissues, the tumor biopsy is digested into a single cell suspension. The cells are segregated into CD45+ (immune cells) and CD45– (tumor cells, stromal cells). Each cell is barcoded and the sequence of each cell generated. This makes it possible to identify the different immune cell populations present in the cancer, to determine the gene expression in those immune cells indicating the activity of the cells, and to determine changes in the number and activity of the immune cells in each population over time. In the future, this is expected to allow the characterization of each step in the immune response in the individual patient. This could facilitate the development of interventions aimed at specific parts of the immune response in need of “help” in the individual.

While canine tumor immunology has lagged behind human tumor immunology, the field is advancing rapidly and will set the stage for high impact studies in dogs to improve immunotherapies across both species.

## Prevention, Early Detection, and Early Intervention, and value of Canine Studies

### Challenges in Cancer Prevention Research

It is well-recognized that prevention of cancer holds the greatest promise for reducing cancer morbidity and mortality, as well as decreasing health care costs (176). This includes primary cancer prevention aimed at stopping cancer from developing, and secondary cancer prevention aimed at detecting cancer early and intervening early to stop its progression. In order to most effectively prevent cancer, crucial information is needed including: (1) environmental and host (genetic) risk factors and gene-environment interactions that lead to cancer, (2) effective methods for screening and early detection, and (3) successful strategies for early intervention (i.e., chemoprevention, diet, drugs, others). This crucial information is very difficult to elucidate in humans. Unlike the case for breast cancer and colon cancer, specific well-defined groups of people with high familial susceptibility for InvUC have not been identified (15). Heritable risks which are likely to exist have not yet been defined, and therefore, cohorts of people who would provide the best study subjects and who would most likely benefit from prevention, early detection, and early intervention have not been established. To further complicate matters, more than half of bladder cancer patients are not aware of any exposures or risk factors that contributed to their cancer (15, 34). And, for people with known carcinogen exposures, the latency period between the carcinogen exposure (i.e., cigarette smoke, specific chemicals) and the development of bladder cancer can extend to decades (15, 33, 34, 177).

Testing prevention strategies can also be challenging. Such research would often require more years of study than would be feasible. If an investigator wanted to test a cancer prevention strategy in humans that would be applied from middle age to the age of typical cancer diagnosis (e.g., from age 40 to age 65 years), the study would require 25 years or more for completion. Clearly, relevant animal models with more compressed life spans are needed for prevention studies in order to select the strategies most likely to be successful in humans. There are many compelling reasons why dogs who have InvUC or who are at risk for developing InvUC can be key models in prevention research.

### Unique Opportunities for Studies in Dogs to Advance Cancer Prevention Research

There are many reasons why dogs are ideally suited to study strategies for prevention, early detection, and early intervention of InvUC (30). The similarities between InvUC in dogs and humans have been detailed in this review. The compressed life span in dogs makes prevention studies feasible in a reasonable length of time. Further, dogs of specific breeds have a much higher risk for developing InvUC than mixed breed dogs as summarized in Table 5 (30).

**Table 5:** Breed-associated risk for InvUC in dogs (30)^*^.

**Breed**	**Number of dogs in that breed in VMDB**	**Number of InvUC cases in that breed**	**OR compared to mixed breed**	**95% confidence intervals**
Mixed breed dog (reference category)	42,777	269	1.0	NA
Scottish Terrier	670	79	21.12	16.23–27.49
Eskimo Dog	225	9	6.58	3.34–12.96
Shetland Sheepdog	2,521	93	6.05	4.76–7.69
West Highland White Terrier	1,234	44	5.84	4.23–8.08
Keeshond	381	10	4.26	2.25–8.07
Samoyed	471	10	3.43	1.81–6.49
Beagle	3236	62	3.09	2.34–4.08
Dalmatian	1253	19	2.43	1.52–3.89

* *Data are summarized from the Veterinary Medical Database (VMDB). The odds ratios (ORs) of InvUC risk compared with the risk in mixed breed dogs are included for breeds with an OR > 2.0 and at least nine cases of InvUC in the breed recorded in the VMDB*.

Scottish Terriers especially stand out for having a 20-fold increased risk for InvUC compared to mixed breed dogs (30). This provides a unique resource to identify existing genes and new genes that have not yet been characterized that contribute to cancer risk and to then assess the expression of those genes in human InvUC patients. Dogs offer a great opportunity to assess environmental risk and gene-environment interactions leading to InvUC. In Scottish Terriers, for example, exposure to lawn chemicals increases the risk of InvUC 7-fold on top of the already existing heritable risk (178). On a more positive front, Scottish Terriers who consume vegetables three times per week in addition to regular dog food have a 70% reduced risk for InvUC (179).

Dogs can also be used to track chemical exposures. With interest arising from the association between lawn chemical exposure and InvUC risk, a study was undertaken to track herbicide exposure and specifically to measure herbicide concentrations in the urine of exposed pet dogs in a community setting. In one study, three chemicals used in lawn care products (2,4-dichlorophenoxyacetic acid, 4-chloro-2-methylphenoxypropionic acid, dicamba) were measured on the grass and in the urine of dogs from 25 households that used lawn chemicals and from eight control households that did not use lawn chemicals (180). Urine samples from the dogs were collected prior to lawn treatment and at 24 and 48 h after lawn treatment. The results were concerning in that after lawn chemicals were applied, the chemicals were detected in the urine of dogs in 19 of 25 treated households. Of even greater concern, chemicals were found in the urine of dogs in 14 of the 25 households before the lawn was treated, and in four of eight control households. This indicated widespread exposure to the chemicals, most likely due in part to chemical drift from other treated areas.

The compressed lifespan of dogs greatly facilitates timely prevention studies. A study in humans from age 40–65 (25 years) could be accomplished in dogs of “similar physiological ages” in 2–4 years. Shorter term interventional strategies could be tested over weeks to several months in dogs. Also, in conducting research to assess the value of a prevention strategy in dogs, it is much more feasible to control other variables (diet, smoking, etc.) than is possible in humans. Regarding establishing the means for cancer screening and early detection, dogs again offer an excellent opportunity because of the compressed life span of dogs, the motivation by pet owners to have cancer detected early in their dog, and the feasibility of non-invasive screening tests and then follow-up confirmatory tests to determine if cancer is or is not present.

## Conclusions

In conclusion, there is strong evidence that dogs with naturally-occurring InvUC can represent a relevant predictive model for InvUC treatment and prevention. Further validation of the canine model could come from parallel human and canine InvUC trials in which the outcome in dogs is predictive of the outcome in humans. Dogs are anticipated to fill an essential niche in cancer drug development and prevention research, and to ultimately transform the outlook for humans (and dogs) facing InvUC.

## Author Contributions

All authors listed have made a substantial, direct and intellectual contribution to the work, and approved it for publication.

### Conflict of Interest

NH acknowledges that he receives an Honoraria—Bladder Cancer Academy, PeerView, PlatformQ Health; that he engages in consulting activity with Bristol-Myers Squibb, AstraZeneca/MedImmune, Pieris Pharmaceuticals, Inovio Pharmaceuticals, Genentech/Roche, Health Advances, Merck, Ferring, Principia, Champions Oncology, Taris Biomedical, Seattle Genetics/Astellas, Incyte, TransMed, Rexahn Pharmaceuticals, CicloMed, Janssen, Celgene, GlaxoSmithKline, Mirati Therpeutics; and that he receives institutional support provided by Genentech/Roche, Merck, Bristol-Myers Squibb, AstraZeneca/MedImmune, Principia Biopharma, Acerta Pharma, Incyte, Seattle Genetics/Astellas, Astex Pharmaceuticals, and Pieris Pharmaceuticals. None of this support or consulting work was related to the content of this manuscript. The remaining authors declare that the research was conducted in the absence of any commercial or financial relationships that could be construed as a potential conflict of interest.
